# Automatic method of analysis of OCT images in assessing the severity degree of glaucoma and the visual field loss

**DOI:** 10.1186/1475-925X-13-16

**Published:** 2014-02-14

**Authors:** Robert Koprowski, Marek Rzendkowski, Zygmunt Wróbel

**Affiliations:** 1 Department of Biomedical Computer Systems, Faculty of Computer Science and Materials Science, Institute of Computer Science, University of Silesia, ul. Będzińska 39, Sosnowiec 41-200, Poland; 2 Individual Specialist Medical Practice, Gliwice, Poland

**Keywords:** Image processing, Measurement automation, OCT, Segmentation, BGA, Biomorphological glaucoma advancement, Glaucoma

## Abstract

**Introduction:**

In many practical aspects of ophthalmology, it is necessary to assess the severity degree of glaucoma in cases where, for various reasons, it is impossible to perform a visual field test - static perimetry. These are cases in which the visual field test result is not reliable, e.g. advanced AMD (Age-related Macular Degeneration). In these cases, there is a need to determine the severity of glaucoma, mainly on the basis of optic nerve head (ONH) and retinal nerve fibre layer (RNFL) structure. OCT is one of the diagnostic methods capable of analysing changes in both, ONH and RNFL in glaucoma.

**Material and method:**

OCT images of the eye fundus of 55 patients (110 eyes) were obtained from the SOCT Copernicus (Optopol Tech. SA, Zawiercie, Poland). The authors proposed a new method for automatic determination of the RNFL (retinal nerve fibre layer) and other parameters using: mathematical morphology and profiled segmentation based on morphometric information of the eye fundus. A quantitative ratio of the quality of the optic disk and RNFL – BGA (biomorphological glaucoma advancement) was also proposed. The obtained results were compared with the results obtained from a static perimeter.

**Results:**

Correlations between the known parameters of the optic disk as well as those suggested by the authors and the results obtained from static perimetry were calculated. The result of correlation with the static perimetry was 0.78 for the existing methods of image analysis and 0.86 for the proposed method. Practical usefulness of the proposed ratio BGA and the impact of the three most important features on the result were assessed. The following results of correlation for the three proposed classes were obtained: cup/disk diameter 0.84, disk diameter 0.97 and the RNFL 1.0. Thus, analysis of the supposed visual field result in the case of glaucoma is possible based only on OCT images of the eye fundus.

**Conclusions:**

The calculations and analyses performed with the proposed algorithm and BGA ratio confirm that it is possible to calculate supposed mean defect (MD) of the visual field test based on OCT images of the eye fundus.

## Introduction

**Figure 1:**
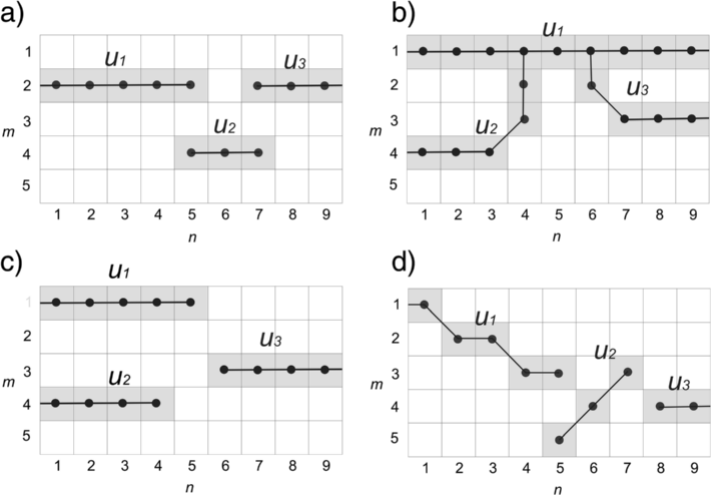
**Diagrams showing problems during recognition of layers in an OCT image of the eye fundus.** The following issues are shown in sequence: **a)** the problem with the gap between *u*_*1*_ and *u*_*3*_ and the impact of the additional new layer *u*_*2*_, **b)** the problem with combining layers *u*_*2*_ and *u*_*3*_ with the layer *u*_*1*_ for 3 pixels (1,4), (1,5) and (1,6), **c)** the difficulty in deciding which layer *u*_*1*_ or *u*_*2*_ should be combined with the layer *u*_*3*_, **d)** the difficulty in deciding whether the layer *u*_*2*_ is the missing piece connecting layers *u*_*1*_ and *u*_*3*_.

Analysis of OCT images of the eye fundus is a technique known for many years which enables to acquire large amounts of information useful for medical diagnosis. Today, there are many known profiled algorithms for the analysis of both successive layers of the eye fundus [1-20] as well as the choroid layer [21]. Depending on the approach and practical implementation, these algorithms operate in the area of texture analysis [1,2], algorithms based on segmentation with Canny method [4], SVM [5], mathematical morphology [6], active contour [7,8], fuzzy algorithms [9], Markov model [10], robust segmentation [11], random contour analysis [12] and others [13-20]. Implementation of these algorithms most commonly occurs in the C, C# programming environment if they are used in commercially available applications, or, for example, in Matlab if they are at the stage of testing and modification [6]. The analysis time of a single OCT image of the fundus is several tens of milliseconds to a few seconds with the most computationally intensive applications [1,17]. In practice, the biggest difficulty is large inter-individual variability and a possible high degree of retinal pathology. This translates into problems with the designation of successive layers of the eye fundus (RNFL–retinal nerve fibre layer, RPE–retinal pigment epithelium, and others) in the following cases (Figure 1):

• The layer (*u*_*1*_, *u*_*3*_) is not continuous - Figure 1a,d,

• The layer (*u*_*2*_, *u*_*3*_) merges with another layer (*u*_*1*_) - Figure 1b,

• One layer ends (*u*_*1*_ or *u*_*2*_) and another one starts (*u*_*3*_) in the same place - Figure 1c.

The mentioned problems can be handled with in a limited way using artificial intelligence (knowledge gained from other images) or anthropometric evidence. In any case, image analysis and the proposed approach to solving such cases are difficult.

From a medical viewpoint, the RNFL thickness is essential in the case of the optic disk analysis. On the basis of its average thickness and further measurements, other parameters such as a cup/disk area ratio or a cup/disk vertical diameter are obtained. These parameters have an influence on the diagnosis of an ophthalmologist as well as the applied methods of treatment.

In the case of glaucoma, it is essential to test the visual field. Its measurement is usually carried out using a static perimeter. A reliable parameter obtained from perimetric data is the average depth of a defect. In many cases, for example, in a number of different additional diseases, it is impossible to obtain this type of measurement or the results obtained are not reliable. Then, it is necessary to search for other methods which will enable to determine the supposed visual field results. One such method is the analysis of OCT images of the eye fundus proposed by the authors. It allows not only to determine the severity degree of glaucoma but also to determine the supposed MD value of visual field test. It is further shown which selected parameters in OCT images correlate, to the greatest extent, with the results obtained from perimetry. Moreover, the new algorithm proposed by the authors and the quantitative ratio of the optic disk condition are described.

### Material

OCT images of the eye fundus of 55 glaucoma patients (110 eyes) were obtained from the SOCT Copernicus (Optopol Tech. SA, Zawiercie, Poland). Patients ranged in age from 37 to 88 years. The obtained images (B-scans) had a resolution of *M* × *N =* 1000 × 600 pixels (where *M* – number of rows, *N –* number of columns) at a colour resolution of 8 bits/pixel. For each patient *I =* 99 B-scans were obtained allowing for full 3D reconstruction of the eye fundus image. At the same time, 110 results were acquired from the static perimeter OCTOPUS 300Series V 6.07c. All data were analysed in a source format. The presented analysis was carried out retrospectively on the basis of data obtained during typical medical examinations. The data were anonymised and the patients were examined in accordance with the Declaration of Helsinki.

## Methods

The method of analysis and processing of OCT images proposed by the authors was preceded by the analysis of the results obtained from conventional methods. This analysis indicates features which should be taken into account when constructing the target image analysis algorithm.

### Selection of the features of the image

**Figure 2:**
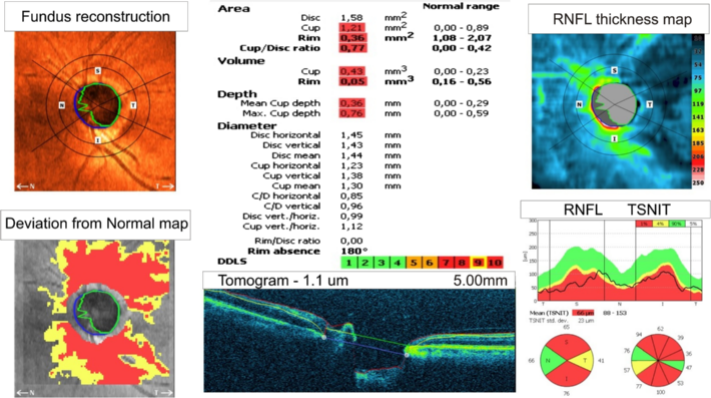
**The result for one patient obtained from the Copernicus tomograph.** The following images can be seen in the corners: fundus reconstruction, a deviation from the normal map, the RNFL thickness map and the graph RNFL TSNIT. In the central part at the bottom there is a sample B-scan of the optic disk. The central part is dedicated to the ratios calculated by the software.

**Figure 3:**
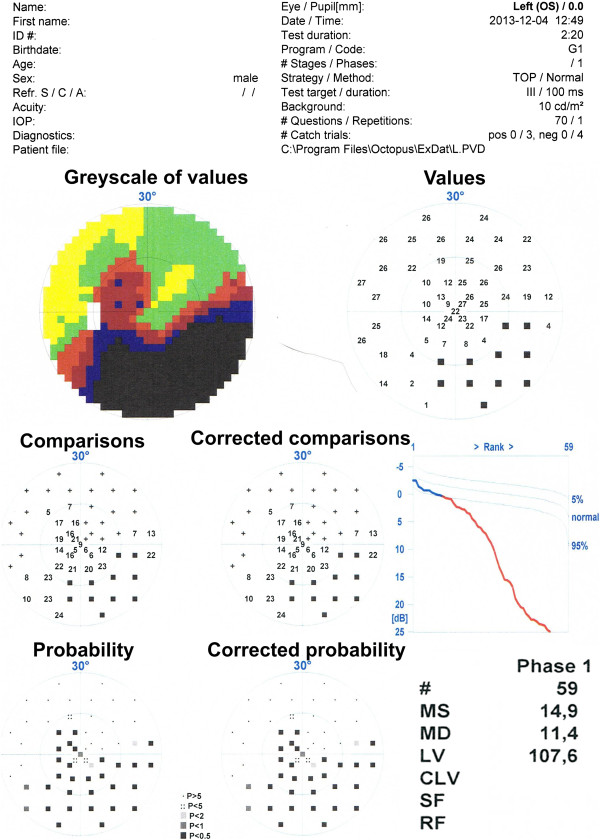
**The result for one patient obtained from the static perimeter.** From the top: data on patient and study, a colour picture of the quality of vision in the 30^O^, numerical data of quality of vision in various fields expressed in decibels: values, comparisons, corrected comparisons, probability and corrected probability. In the lower right corner there are two most important parameters, namely MD (Mean Defect) and LV (Loss Variance). The analysis did not take into account LV value, because there was no significant correlation between LV and any of the OCT parameters.

**Figure 4:**
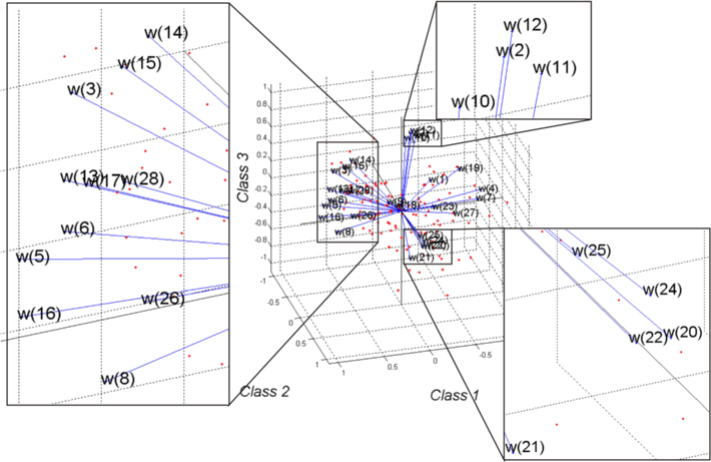
**The result of factor analysis of 28 features and their division into 3 classes.** Each of the 28 features is represented in this plot by a vector, and the direction and length of the vector indicate how each feature depends on the underlying factors (class). The orthogonal nature of the majority of the features, in particular *w*(16), *w*(22) to *w*(25) and *w*(1), *w*(2) *w*(12) as well as *w*(11), is clearly visible. Individual observations are marked in red. On the right side in the corners the characteristic areas have been enlarged.

As stated in the previous section, a visual field test was performed on 55 glaucoma patients. The features of the eye fundus were measured using a Copernicus tomograph. The results obtained in the software available for these devices are shown in Figure 2 and Figure 3. The features obtained on this basis are presented in Table 1. Examples of values for each feature for the first 10 patients are shown in Table 2. Each of these characteristics will be further, for simplicity, denoted by the symbol “*w*”. In this case, the features from *w*(1) to *w*(28) were acquired from the tomograph, and *w*(29) and *w*(30) from the perimeter. The parameters from *w*(1) to *w*(30) were further subjected to exploratory factor analysis in order to detect their cross correlation and to remove those features which are statistically insignificant. For this purpose, the following items were designated in succession: correlation of features *w*, eigenvalues of the correlation matrix, the number of classes using the Kaiser’s and Cattell’s criteria, rotation of the coordinate system, correlation of individual features with the newly created classes. The results (for the whole group of patients) are shown in Table 3 whereas the correlation chart of various features with three classes are shown in Figure 4. The analysis of Table 3 and Figure 4 suggests several conclusions useful for further analysis:

**Table 1:** Image features obtainable with the known software of the tomograph and the static perimeter

***Tomograph***		***Tomograph***	
**Feature – symbol**	**Description**	**Feature – symbol**	**Description**
*w*(1)	Patient age	*w*(17)	C/D diameter vertically
*w*(2)	Disk area	*w*(18)	Disk vertical/horizontal
*w*(3)	Cup area	*w*(19)	Cup vertical/horizontal
*w*(4)	Rim area	*w*(20)	RNFL mean thickness
*w*(5)	Cup/Disk area	*w*(21)	RNFL thickness temporal
*w*(6)	Cup volume V	*w*(22)	RNFL thickness top
*w*(7)	Rim volume V	*w*(23)	RNFL thickness nasal
*w*(8)	Mean depth	*w*(24)	RNFL thickness bottom
*w*(9)	Maximum depth	*w*(25)	SD
*w*(10)	Disk diameter horizontally	*w*(26)	DDLS
*w*(11)	Disk diameter vertically	*w*(27)	R/D ratio
*w*(12)	Mean disk diameter	*w*(28)	RIM abs
*w*(13)	Cup diameter horizontally	***Perimeter***	
*w*(14)	Cup diameter vertically	*w*(29)	MD
*w*(15)	Cup mean diameter	*w*(30)	LV
*w*(16)	C/D diameter horizontally		

**Table 2:** Sample results for the first 10 patients obtained from the tomograph and perimeter

**Feature/Patient**	**1**	**2**	**3**	**4**	**5**	**6**	**7**	**8**	**9**	**10**
*w*(1)	83	53	53	53	53	79	79	63	63	67
*w*(2)	2.26	1.49	1.88	1.90	1.87	2.01	2.19	1.83	1.49	1.98
*w*(3)	0.68	1.32	1.530	1.88	1.85	1.96	0.98	1.50	1.24	1.28
*w*(4)	1.58	0.17	0.34	0.02	0.02	0.05	1.21	0.33	0.26	0.70
*w*(5)	0.30	0.89	0.82	0.99	0.99	0.98	0.45	0.82	0.83	0.64
*w*(6)	0.12	0.67	0.73	0.83	0.79	0.46	0.05	0.71	0.46	0.39
*w*(7)	0.22	0.01	0.02	0	0	0.01	0.07	0.02	0.02	0.07
*w*(8)	0.17	0.50	0.47	0.44	0.43	0.24	0.05	0.47	0.37	0.31
*w*(9)	0.43	0.93	0.93	0.80	0.91	0.55	0.20	0.93	0.85	0.68
*w*(10)	1.57	1.40	1.58	1.53	1.47	1.42	1.71	1.47	1.34	1.52
*w*(11)	1.80	1.38	1.53	1.52	1.52	1.61	1.52	1.61	1.42	1.61
*w*(12)	1.69	1.39	1.55	1.52	1.50	1.52	1.61	1.54	1.38	1.56
*w*(13)	0.68	1.31	1.35	1.52	1.47	1.42	1.16	1.33	1.23	1.12
*w*(14)	1.23	1.28	1.48	1.61	1.61	1.71	1.33	1.42	1.23	1.42
*w*(15)	0.96	1.29	1.42	1.57	1.54	1.56	1.25	1.38	1.23	1.27
*w*(16)	0.43	0.94	0.86	1.00	1.00	1.00	0.68	0.91	0.92	0.74
*w*(17)	0.68	0.93	0.97	1.06	1.06	1.06	0.88	0.88	0.87	0.88
*w*(18)	1.14	0.98	0.97	0.99	1.03	1.13	0.89	1.10	1.06	1.06
*w*(19)	1.81	0.97	1.09	1.06	1.09	1.20	1.14	1.07	1.00	1.27
*w*(20)	93	68	88	60	67	85	80	65	68	88
*w*(21)	60	45	45	47	57	86	67	59	54	70
*w*(22)	104	109	88	59	67	103	100	80	80	116
*w*(23)	75	47	62	52	50	57	87	60	58	76
*w*(24)	106	48	88	71	80	84	62	57	67	75
*w*(25)	25	33	24	19	18	28	23	17	20	25
*w*(26)	4	9	7	10	10	8	6	7	9	7
*w*(27)	0.11	0	0	0	0	0	0	0	0	0
*w*(28)	0	150	57	319	325	326	27	68	109	70
*w*(29)	0.1	6.3	2.8	23.6	24.7	22.4	8.5	2.5	0.2	8.5
*w*(30)	4.6	58.8	6.8	58.5	46.2	40.9	125.6	38.6	6.30	151

**Table 3:** The results (for the whole group of patients) obtained from factor analysis for the three classes (bold >0.5, underlined >0.7)

***Feature/Class***	**Class 1**	**Class 2**	**Class 3**
*w*(16)	**0.84**	−0.12	−0.01
*w*(13)	**0.77**	−0.05	0.33
*w*(6)	**0.75**	−0.10	0.17
*w*(5)	**0.75**	−0.34	−0.001
*w*(8)	**0.71**	0.018	−0.14
*w*(3)	**0.69**	−0.19	0.46
*w*(15)	**0.56**	−0.21	0.49
*w*(17)	**0.53**	−0.46	0.08
*w*(14)	0.46	−0.28	**0.51**
*w*(28)	0.42	−0.50	0.04
*w*(26)	0.42	−0.39	−0.19
*w*(9)	0.13	−0.02	0.11
*w*(21)	0.11	0.72	−0.18
*w*(18)	0.07	0.04	0.09
*w*(22)	0.04	**0.94**	0.05
*w*(20)	0.02	**1.00**	0.08
*w*(24)	0.02	**0.93**	0.11
*w*(25)	−0.001	**0.67**	0.039
*w*(10)	−0.014	0.11	**0.90**
*w*(2)	−0.05	0.15	**0.97**
*w*(12)	−0.08	0.10	**0.97**
*w*(11)	−0.13	0.07	**0.90**
*w*(23)	−0.19	**0.54**	0.28
*w*(1)	−0.40	−0.31	0.15
*w*(27)	−0.49	0.31	0.05
*w*(19)	**−0.72**	−0.33	0.22
*w*(7)	**−0.74**	0.29	0.18
*w*(4)	**−0.76**	0.32	0.30

Class 1 significantly (above 0.5) correlates with the features *w*(16), *w*(13), *w*(6), *w*(5), *w*(8), *w*(3), *w*(15) and *w*(17) and is in a negative correlation with the features *w*(19), *w*(7) and *w*(4). It means that the cup/disk diameter and the cup diameter are a representative of the class 1. The ratio of the cup vertical/horizontal and the area and volume of the rim are in the inverse correlation.

Class 2 significantly correlates with the features *w*(22), *w*(20), *w*(24), *w*(25), *w*(23). These features are responsible for the RNFL parameters.

Class 3 correlates with the features *w*(10), *w*(2), *w*(12), *w*(11) and *w*(14) which are responsible for the parameters of the disk diameter in the vertical and horizontal axes.

Analysis of this division into classes, their correlation with the results obtained from perimetry and interpretation of individual features allow to specify the features of OCT images necessary for further analysis. The particularly important features of OCT image are: disk diameter and its relation to cup as well as the RNFL parameters in the area. This information is the basis for the design of a profiled image analysis algorithm, correlation analysis with the results of perimetry and for suggesting a new quantitative ratio of the optic disk condition.

### Pre-processing

**Figure 5:**
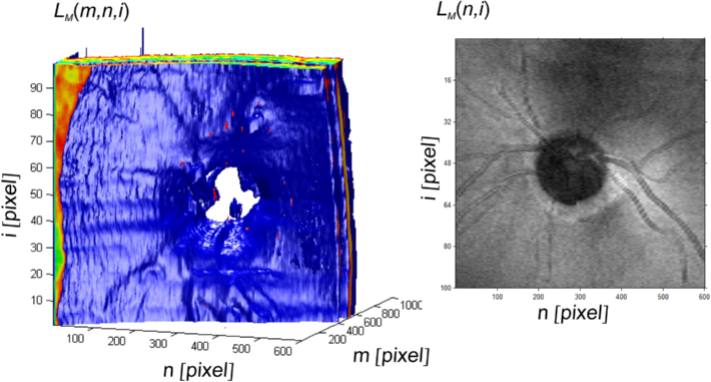
**The result of 3D reconstruction of the image *L***_***M***_**(*m,n,i*) and the image *L***_***M***_**(*n,i*).** The first image is shown in an artificial colour palette in the Cartesian coordinate system. The characteristic layers RNFL and RPE, optic disk, and the distribution of blood vessels are visible. This image is the basis for further analysis and verification of practical usefulness of the algorithm proposed by the authors. The other image is the brightness of the pixels in the matrix system with *i* rows and *n* columns showing the exact location of the vessels and the optic disks.

Pre-processing of images is related to automatic unpacking of the source file with the extension *.oct and thus acquiring images in the *.jpg format and calibration data. These data mainly include information recorded by the tomograph in the info.ini file, for example, the position of subsequent B-scans in space, the numbers of A-scans and B-scans, date of test, the patient data and others. The sequence of images *L*_*GRAY*_(*m,n,i*) obtained on this basis is further subjected to filtration with a median filter whose mask *h*_*1*_ is sized *M*_*h1*_ × *N*_*h1*_ *× I*_*h1*_*=* 3 × 3 × 3 pixels. The mask size was chosen arbitrarily taking into account the size of distortions and artefacts entering the optical path. The filtered image *L*_*M*_(*m,n,i*) (Figure 5) is subjected to further preliminary transformations. These include determination of the RNFL.

### Processing – RPE, ILM, RNFL, cup, disk

In order to determine the RNFL, the RPE layer (retinal pigment epithelium) is pre-determined automatically. The RPE layer is the most characteristic and the brightest layer in an OCT image, therefore, it is used to determine the target RNFL. Additionally, the RPE is used to determine the disk boundary. For this purpose, every *n*-th column of the image *L*_*M*_, each A-scan, is analysed. Analogously to the method described in [21,22], the position of the maximum brightness for each column is pre-determined, i.e.:

(1) $$L_{\mathrm{RPE}}\left(n,i\right)=\arg\left(\max_{m\in \left(1,M\right)}\left(L_{M}\left(m,n,i\right)\right)\right)$$

where:

*m,n* – coordinates of the rows and columns of the matrix *m*∈(1,*M*) and *n*∈(1,*N*).

The formula (1) can be directly applied only when for all the analysed rows of each column there is only one maximum brightness value. As shown in [6], in practice it occurs in about 80% of the cases analysed at a resolution of 8 bits per pixel. On this basis, two essential elements are calculated: disk and ILM layer. The disk location, the image *L*_*disk*_, is here calculated as the area:

(2) $$L_{\mathrm{disk}}\left(n,i\right)=\left\{\begin{array}{ccc} 1 & \mathrm{if} & L_{\mathrm{RPE}}\left(n,i\right)>p_{m}\cdot \underset{n,i}{\mathrm{mean}}\left(L_{\mathrm{RPE}}\left(n,i\right)\right)\\ 0 & \mathrm{other} & \; \end{array}\right)$$

where: mean – mean value,

*p*_*m*_ – threshold equal 2/3.

The value of 2/3 (*p*_*m*_) was chosen arbitrarily and is associated with the maximum acceptable shift of the RPE layer in the area of the optic disk. The other element, namely the ILM layer area, was calculated as the area from the first pixel of each row to *L*_*RPE*_(*n,i*). It is very easy to perform binarization in this area using Otsu method [23], thus setting the binarization threshold *p*_*r*_. The resulting binary image *L*_*B*_(*m,n,i*) is:

(3) $$L_{B}\left(m,n,i\right)=\left\{\begin{array}{ccc} 1 & \mathrm{if} & \left(L_{M}\left(m,n,i\right)>p_{r}\right)\wedge \left(m\leq L_{\mathrm{RPE}}\left(n,i\right)\right)\;\\ 0 & \mathrm{others} & \; \end{array}\right)$$

Based on the binary image *L*_*B*_(*m,n,i*), the edge of the ILM layer was defined as:

(4) $$L_{S}\left(m,n,i\right)=\left\{\begin{array}{ccc} m & \mathrm{if} & L_{B}\left(m,n,i\right)=1\\ M & \mathrm{other} & \; \end{array}\right)$$

(5) $$L_{\mathrm{ILM}}\left(n,i\right)=\min_{m\in \left(1,M\right)}\left(L_{S}\left(m,n,i\right)\right)$$

The waveform of *L*_*ILM*_(*n,i*) and the RPE boundary, i.e. *L*_*RPE*_(*n,i*), are the basis for further analysis of the RNFL layer and cup area. The area between the layers ILM and RPE is further subjected to an operation which involves determination of the edge using two orthogonal Prewitt masks *h*_*h*_ and *h*_*v*_ sized 3 × 3 pixels. Filtration results in the image *L*_*G*_(*m,n,i*), i.e.:

(6) $$L_{G}\left(m,n,i\right)=\left\{\begin{array}{ccc} \sqrt{L_{\mathrm{Gh}}{\left(m,n,i\right)}^{2}+L_{\mathrm{Gv}}{\left(m,n,i\right)}^{2}} & \mathrm{if} & \left(m>L_{\mathrm{ILM}}\left(n,i\right)\right)\wedge \left(m<L_{\mathrm{RPE}}\left(n,i\right)\right)\\ 0 & \mathrm{other} & \; \end{array}\right)$$

where: *L*_*Gh*_(*m,n,i*) and *L*_*Gv*_(*m,n,i*) are the result of convolution of the image *L*_*M*_(*m,n,i*) with the masks *h*_*h*_ and *h*_*v.*_

The RNFL is characterized by a contour visible in each column of the image *L*_*M*_(*m,n,i*) as a transition from a light area (below the ILM) to a dark one (the other layers). It is therefore the first edge below the ILM for *L*_*G*_(*m,n,i*) > 0, i.e. (analogously to (4), (5)):

(7) $$L_{R}\left(m,n,i\right)=\left\{\begin{array}{ccc} m & \mathrm{if} & \left(L_{G}\left(m,n,i\right)>0\right)\wedge \left(m>L_{\mathrm{ILM}}\left(n,i\right)\right)\wedge \left(m<L_{\mathrm{RPE}}\left(n,i\right)\right)\\ M & \mathrm{other} & \; \end{array}\right)$$

(8) $$L_{\mathrm{RNFL}}\left(n,i\right)=\min_{m\in \left(1,M\right)}\left(L_{R}\left(m,n,i\right)\right)$$

**Figure 6:**
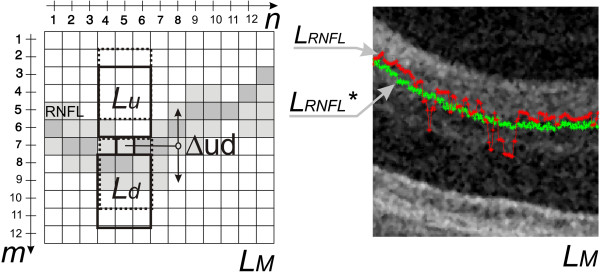
**The results of the dynamic adjustment of the contour position.** On the left side, the idea of the dynamic contour position adjustment has been shown. The difference between the mean values in the areas *L*_*u*_ and *L*_*d*_ provides information about the correct position of the point. The greatest difference between the areas *L*_*u*_ and *L*_*d*_ is searched for in a selected area. The right side shows an example of the input waveform of *L*_*RNFL*_(*n,i*) in red and the result of the position adjustment of *L*_RNFL_^*^(*n,i*) in green.

The protection (*m > L*_*ILM*_(*n,i*))∧(*m < L*_*RPE*_(*n,i*)) is additional and prevents detection of the RNFL on the edge of the analysed object with the background. The resulting edge has a number of disadvantages. The biggest one is the lack of continuity caused by both occurring shadows as well as insufficient contrast in the place of its detection. For such cases, the method of active edge correction was suggested. This method involves adjustment of the position of the layer *L*_*RPE*_(*n,i*). For each point of the layer *L*_*RPE*_(*n,i*), the mean brightness value above and below *L*_*RPE*_(*n,i*) in the areas *L*_*u*_ and *L*_*d*_ is determined - Figure 6. The new corrected position of the layer *L*_RPE_^*^(*n,i*) is determined as the maximum difference between the average values in the areas *L*_*u*_(Δ*ud,n,i*) and *L*_*d*_(Δ*ud,n,i*). These areas are shifted in the column axis in the range Δ*ud =* ±med(|*L*_*ILM*_(*n,i*)-*L*_*RPE*_(*n,i*)|). The adjusted position of the RNFL is therefore calculated as:

(9) $$L_{\mathrm{RNFL}}^{*}\left(n,i\right)=\underset{m}{\arg}\left(\max_{\Delta \mathrm{ud}}\left(\left|L_{u}\left(\mathrm{\Delta ud},n,i\right)-L_{d}\left(\mathrm{\Delta ud},n,i\right)\right|\right)\right)$$

The resulting new position of the layer *L*_RNFL_^*^(*n,i*) is the basis for further analysis. On the basis of the positions of the ILM and RPE layers, i.e.: *L*_*ILM*_(*n,i*), *L*_*RPE*_(*n,i*), the cup area *L*_*cup*_(*n,i*) is determined. This area is calculated as the intersection of the boundary of *L*_*disk*_(*n,i*) at the offset height equal to 150 μm with the layer *L*_*ILM*_(*n,i*).

As evident from the exploratory factor analysis presented in the previous section, apart from the RNFL thickness, also limitation of the analysis area - ROI (region of interest) is vital. The ROI ranges from 2∙*r*_*disk*_ to 3∙*r*_*disk*_ in an OCT image and in order to determine it, it is necessary to determine the position of coordinates of the centre (*n*_*d0*_*,i*_*d0*_), i.e.:

(10) $$n_{d0}=\frac{\sum_{i=1}^{I}\sum_{n=1}^{N}\left(L_{\mathrm{disk}}\left(n,i\right)\cdot n\right)}{\sum_{i=1}^{I}\sum_{n=1}^{N}L_{\mathrm{disk}}\left(n,i\right)}$$

(11) $$i_{d0}=\frac{\sum_{i=1}^{I}\sum_{n=1}^{N}\left(L_{\mathrm{disk}}\left(n,i\right)\cdot i\right)}{\sum_{i=1}^{I}\sum_{n=1}^{N}L_{\mathrm{disk}}\left(n,i\right)}$$

The value of *r*_*disk*_ is the average radius calculated from the area of a circle:

(12) $$r_{\mathrm{disk}}=\sqrt{\frac{\sum_{i=1}^{I}\sum_{n=1}^{N}L_{\mathrm{disk}}\left(n,i\right)}{\pi }}$$

The calculation of the centre of gravity is followed by the conversion of the ROI to the polar coordinate system, i.e.:

(13) $$\theta =\mathrm{atan}2\left(n-n_{d0},i-i_{d0}\right)$$

(14) $$\rho =\sqrt{{\left(n-n_{d0}\right)}^{2}+{\left(i-i_{d0}\right)}^{2}}$$

**Figure 7:**
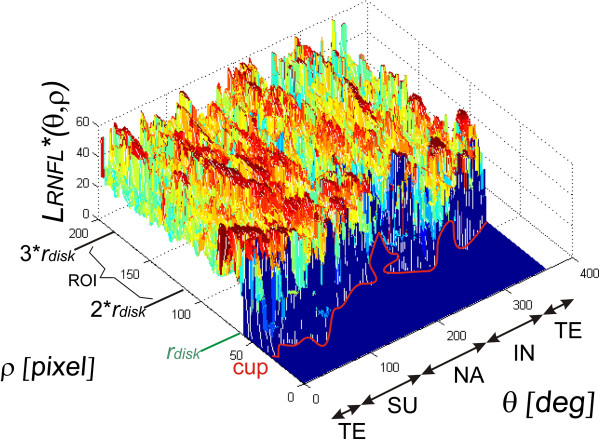
**The results of the region of interest (ROI) separation in the polar coordinate presented in Cartesian coordinate system.** The present region of interest is the basis for further analysis related to the determination of the features associated with the area change in the RNFL thickness and the percentage of the area cup/disk ratio. These features are the basis for determining their correlation with the results of perimetry. The areas TE, SU, NA, IN denoting the following areas respectively: temporal, superior, nasal, inferior have been marked on the axis *θ*. The cup boundaries are marked in red and disk boundaries in green.

**Figure 8:**
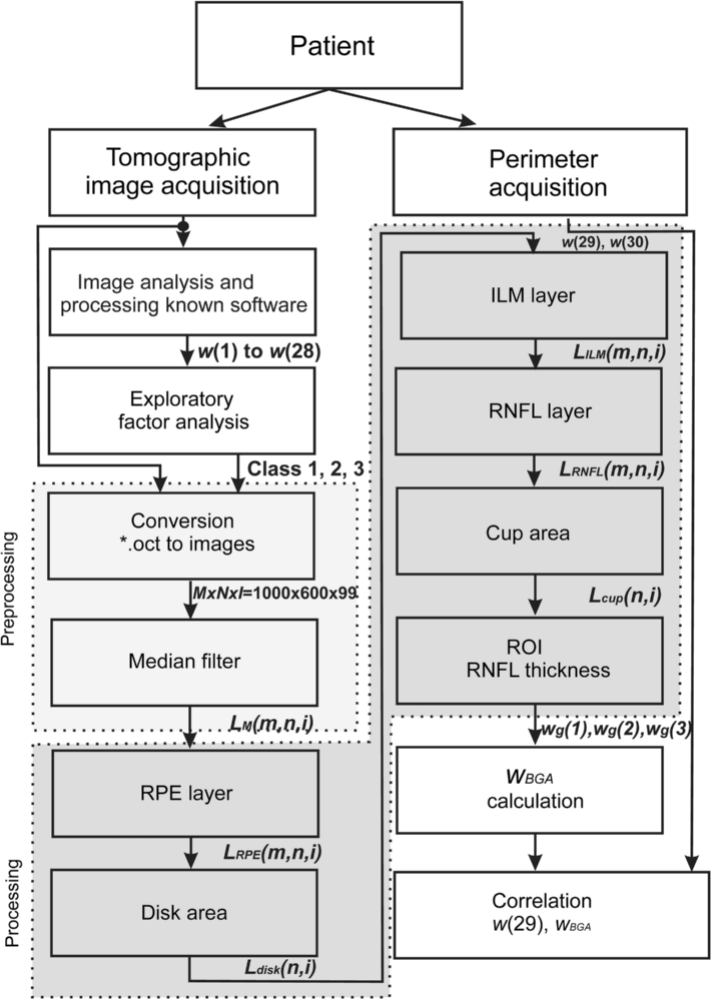
**Block diagram of the algorithm proposed by the authors.** The patient undergoes a fundus examination with the use of a tomograph and their visual field is measured with a perimeter. Then, the significance and correlation of the features obtained are calculated, thereby forming three classes. They are the basis for proposing a new analysis algorithm. After automatic determination of the layers RPE, ILM, RNFL and the cup and disk, the features *w*_*g*_(1), *w*_*g*_(2) and *w*_*g*_(3) are determined. In the final stage, the proposed BGA ratio and correlation with results obtained from perimetry are calculated.

The function *atan2* returns *θ* the same size as *n* and *i* containing the element-by-element, four-quadrant inverse tangent (arctangent) of the real parts of *n* and *i*. The obtained results, the ROI in the polar coordinate system *L*_RNFL_^*^(*θ,ρ*), are shown in Figure 7. The proposed algorithm is shown globally in a block diagram form in Figure 8. On the basis of the presented ROI, the following results (features) were obtained: the disk diameter and its relation to the cup and RNFL parameters. Their thorough analysis and correlation with the results from perimetry are presented in the next section.

## Results

The obtained result *L*_RNFL_^*^(*θ,ρ*), the ROI in the polar coordinate system (Figure 7), is the basis for further analysis. Based on the presented exploratory factor analysis and the proposed method of image analysis and processing, the features representing different classes (Figure 4, Table 3), associated with *L*_RNFL_^*^(*θ,ρ*), were formulated in the following way:

*w*_*g*_(1) – the relative difference in the positions of centres of gravity calculated as:

(15) $$w_{g}\left(1\right)=\frac{\sqrt{{\left(n_{d0}-n_{c0}\right)}^{2}+{\left(i_{d0}-i_{c0}\right)}^{2}}}{r_{\mathrm{disk}}}$$

where:

*n*_*d0*_*, i*_*d0*_ – coordinates of the centre of gravity of the disk,

*n*_*c0*_*, i*_*c0*_*–* coordinates of the centre of gravity of the cup,

*w*_*g*_(2) *–* relative, inverse, minimum distance between the boundaries of the cup and disk i.e.:

(16) $$w_{g}\left(2\right)=1-\frac{r_{\min}}{r_{\mathrm{disk}}}$$

where: *r*_*min*_ – minimum distance between the boundaries of the cup and disk.

*w*_*g*_(3) – RNFL thickness deviation in the ROI (*L*_*RNFL*_^*ROI*^(*θ*)) for particular areas: temporal (TM), superior (SU), nasal (NA), interior (IN) from the standard thickness changes:

(17) $$w_{g}\left(3\right)=\frac{1}{360}\sum_{\theta }\left|\frac{L_{\mathrm{RNFL}}^{\mathrm{ROI}}\left(\theta \right)-L_{\mathrm{RNFL}}^{\mathrm{WZ}}\left(\theta \right)}{L_{\mathrm{RNFL}}^{\mathrm{WZ}}\left(\theta \right)}\right|$$

where: *L*_*RNFL*_^*WZ*^(*θ*) – the reference value of the RNFL thickness (the graph highlighted in green in Figure 2).

The mentioned features are components of the proposed biomorphological glaucoma advancement ratio (BGA) which is defined as:

(18) $$w_{\mathrm{BGA}}=\frac{1}{3}\cdot \left(w_{g}\left(1\right)+w_{g}\left(2\right)+w_{g}\left(3\right)\right)$$

After value substitution and simplification:

(19) $$w_{\mathrm{BGA}}=\frac{\sqrt{{\left(n_{d0}-n_{c0}\right)}^{2}+{\left(i_{d0}-i_{c0}\right)}^{2}}+r_{\mathrm{disk}}-r_{\min}}{3\cdot r_{\mathrm{disk}}}+\frac{w_{g}\left(3\right)}{3}$$

**Figure 9:**
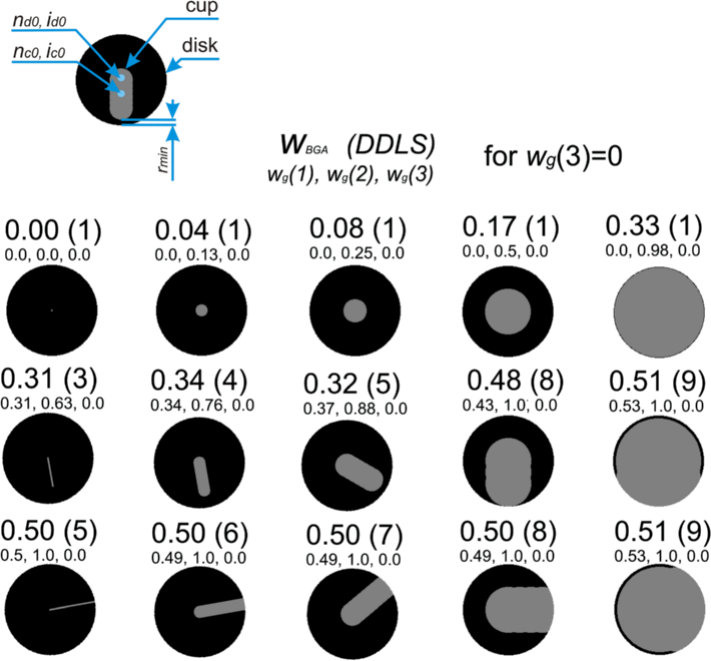
**The impact of cup arrangement with respect to the disk on the values *w***_***g***_**and *w***_***BGA***_**.** A summary of various typical cup arrangements relative to the disk and the effect of their size on the features *w*_*g*_(1), *w*_*g*_(2) and *w*_*g*_(3) as well as *w*_*BGA*_ and, in addition, the values of the DDLS, have been shown. A change in the position of the cup centre of gravity relative to the disk is expressed by the feature *w*_*g*_(1). In turn, the feature *w*_*g*_(2) is a measure of the centricity of the cup edge distribution with respect to the disk. *w*_*g*_(3) is the mean value of the RNFL thickness deviation from the standard.

Figure 9 presents a list of various types of cup arrangements relative to the disk and the impact of their sizes on the values of the features *w*_*g*_(1), *w*_*g*_(2) and *w*_*g*_(3) as well as *w*_*BGA*_. In addition, for comparison, the values of the DDLS (Disk Damage Likelihood Scale) were also shown [24]. A change in the position of the centre of gravity of the cup relative to the disk is expressed by the feature *w*_*g*_(1). The closer the centres of gravity of the cup are to each other, the closer the feature *w*_*g*_(1) is to zero. If the centres of gravity of the cup and disk are far apart, the extreme position of the cup is on the edge of the disk, then the value of *w*_*g*_(1) is close to one. In turn, the feature *w*_*g*_(2) is a measure of centricity of the distribution of cup edges relative to the disk. When the edge of the cup is substantially spaced from the edge of the disk, the value of *w*_*g*_(2) is close to zero. When the edges of the cup and the disk overlap in any area, then the value of *w*_*g*_(2) is close to one. The situation is similar in the case of advanced glaucoma where *r*_*min*_ is close to zero and *w*_*g*_(2) is close to one. A change in the value of *w*_*g*_(3) is related to a deviation with respect to the standard waveform of the RNFL thickness (Figure 2). The greater this difference is, the larger the value of *w*_*g*_(3) is, and vice versa. The features *w*_*g*_(1), *w*_*g*_(2) and *w*_*g*_(3) are summed up and thus determine the ratio *w*_*BGA.*_ The ratio *w*_*BGA*_ reaches values close to one if all three features *w*_*g*_ indicate pathology. In the case of the right features *w*_*g*_, the ratio *w*_*BGA*_ is equal to zero.

Based on the proposed ratio *w*_*BGA*_, its correlation with the results of perimetry was determined. In addition, it was compared with the results obtained only on the basis of known software (features *w*(1) to *w*(29)). The following results were obtained:

(20) $$w\left(29\right)=24\cdot w_{\mathrm{BGA}}-9.34$$

**Figure 10:**
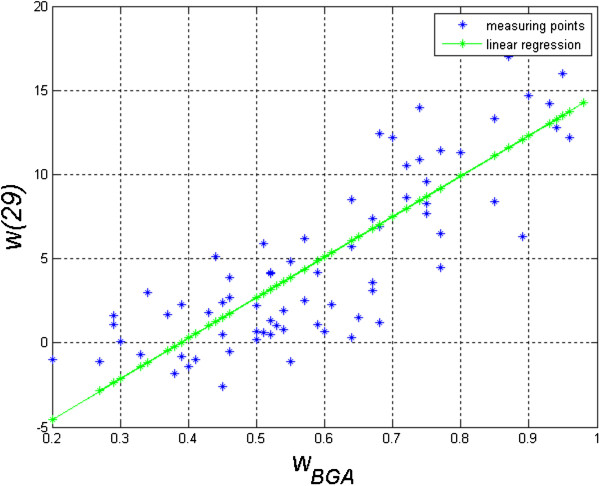
**Graph of the dependence of *w*(29) as a function of changes in the ratio *w***_***BGA***_**.** The graph shows in blue the results obtained for individual patients. The result of linear regression is marked in green. The function describing the dependence of *w*(29) on *w*_*BGA*_ takes the following form: *w*(29) = 24⋅*w*_*BGA*_**–**9.34. On this basis, when the ratio *w*_*BGA*_ is calculated, it is possible to calculate the expected result of perimetry.

• Correlation of classes 1, 2, 3 (obtained from features *w*(1) to *w*(29)) with the results obtained from perimetry is 0.78,

• Correlation of the proposed ratio *w*_*BGA*_ referred to the described method of image analysis and processing with the results of perimetry is 0.86 – Figure 10. The function describing the dependence of *w*(29) (denoting the MD value from perimetry) on *w*_*BGA*_ takes the following form:

The difference in the correlation results stems from the profiled method of image analysis and processing and the measurement methodology of the described issue.

## Summary

The paper presents a new methodology for image analysis and processing profiled for the optic disk analysis with a particular emphasis on its correlation with static perimetry. The calculations and analyses performed with the proposed algorithm confirm the possibility of prediction of the visual field result based on OCT images of the eye fundus. The analysis time of a single OCT image of the eye fundus with the use of the proposed algorithm on the computer Core i5 CPU M460 @ 2.5GHz 4GB RAM does not exceed 0.2 s. Additionally, a new ratio reliable with respect to the quantitative assessment of the optic disk condition was proposed. On its basis, a comparison of the results of the correlation between the known methods and the methodology described in this paper was made.

The present paper does not exhaust this interesting subject. The proposed method of image analysis and processing is one of the possible approaches to solving this type of task. Certainly, other methods of image analysis and processing can be used here [25,26] such as texture analysis [27,28] or other methods applied to solving similar issues [29-31].

In further studies, the authors intend to test practical usefulness of the proposed algorithm and the ratio *w*_*BGA*_. The results obtained for large inter-individual variability (diversity of shapes and topology of the optic disk), repeatability of the data and the impact of technological diversity and device parameters (tomograph and perimeter) on the result are particularly vital here.

## Abbreviations

AMD: Age-related macular degeneration; ROI: Region of interest; RNFL: Retinal nerve fiber layer; TE: Temporal; SU: Superior; NA: Nasal; IN: Inferior; BGA: Biomorphological glaucoma advancement; MD: Mean defect; LV: Loss variance.

## Competing interests

The authors declare that they have no competing interests.

## Authors’ contributions

RK suggested the algorithm for image analysis and processing, implemented it and analyzed the images. MR, ZW performed the acquisition of the tomography images and consulted the obtained results. All authors have read and approved the final manuscript.
